# Supplementary material for: Localized surface plasmon resonance inflection points for improved detection of chemisorption of 1-alkanethiols under total internal reflection scattering microscopy
Source: Sci Rep. 2021 Jun 18;11:12902. doi: 10.1038/s41598-021-92410-w (PMC8213723; doi:10.1038/s41598-021-92410-w)
Supplement: Supplementary file 1 — Supplementary Information 1. [file 41598_2021_92410_MOESM1_ESM.doc]

**Supplementary Information**

**Localized Surface Plasmon Resonance Inflection Points for Improved Detection of Chemisorption of 1-alkanethiols under Total Internal Reflection Scattering Microscopy**

Kyeong Rim Ryu,^a,#^ Geun Wan Kim,^a,#^ and Ji Won Ha^a,b*^

^a^Advanced Nano-Bio-Imaging and Spectroscopy Laboratory, Department of Chemistry, University of Ulsan, 93 Daehak-ro, Nam-gu, Ulsan 44610, Republic of Korea

^b^Energy Harvest-Storage Research Center (EHSRC), University of Ulsan, 93 Daehak-ro, Nam-gu, Ulsan 44610, Republic of Korea

^#^These authors contributed equally to this work.

^*^To whom correspondence should be addressed.

**J. W. Ha**

Phone: +82-52-712-8012

Fax: +82-52-712-8002

E-mail: jwha77@ulsan.ac.kr

This document contains supplementary figures (Figs. S1 to S5).

**Supplementary Figures**


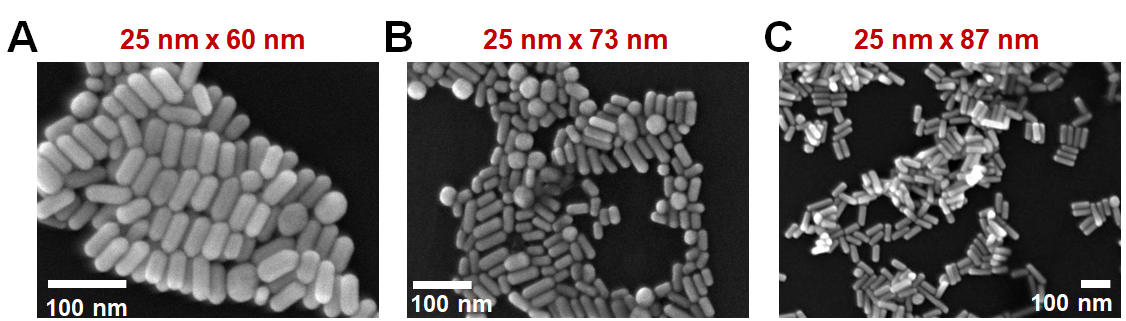


**Figure S1.** **(A)** SEM image of AuNRs with a AR of 2.4 (25 nm × 60 nm). **(B)** SEM image of AuNRs with a AR of 2.9 (25 nm × 73 nm). **(C)** SEM image of AuNRs with a AR of 3.5 (25 nm × 87 nm).

**
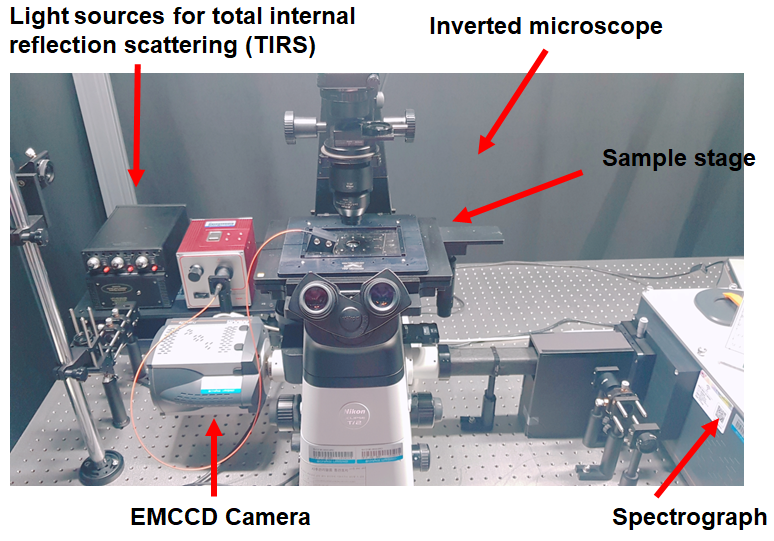
**

**Figure S2.** A photograph to show the experimental setup for single-particle TIRS microscopy and spectroscopy.


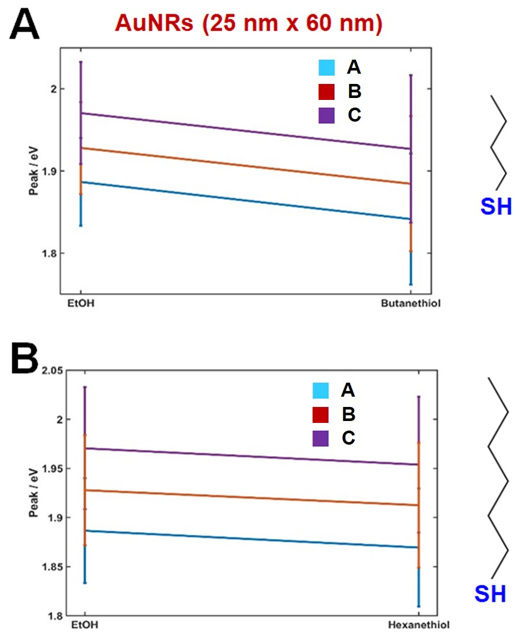


**Figure S3.** **(A)** Peak energy plotted against the chemical adsorption of 1-butanethiol for points A, B, and C in single AuNRs (25 nm × 60 nm, AR=2.4). **(B)** Peak energy plotted against the chemical adsorption of 1-hexanethiol for points A, B, and C in single AuNRs (25 nm × 60 nm, AR=2.4).

**
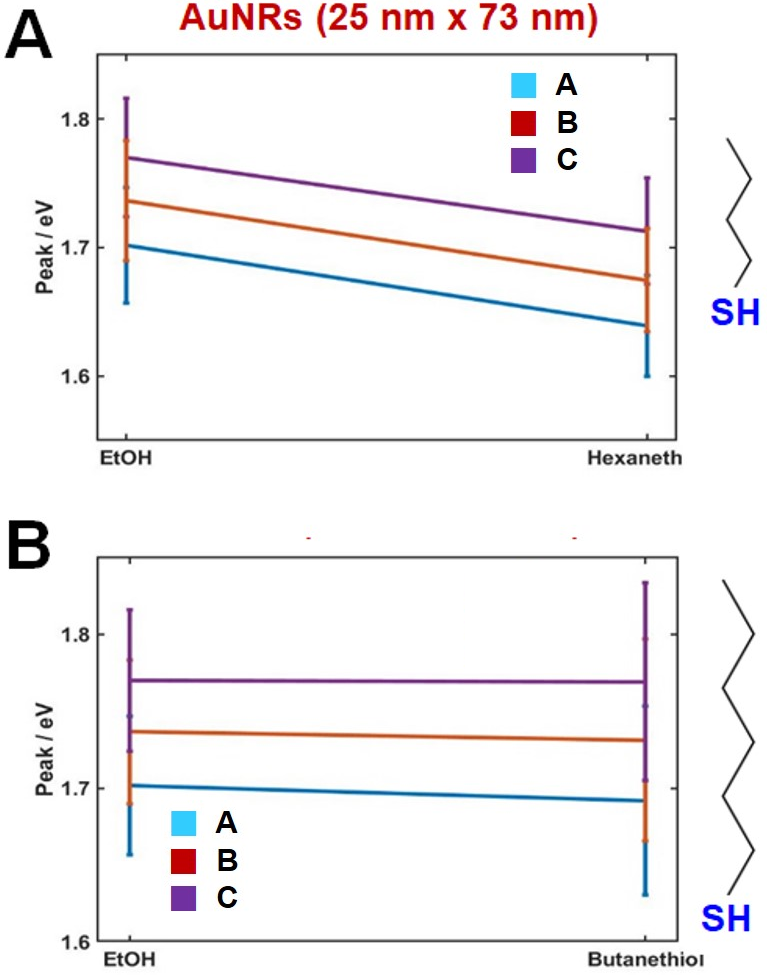
**

**Figure S4.** **(A)** Peak energy plotted against the chemical adsorption of 1-butanethiol for points A, B, and C in single AuNRs (25 nm × 73 nm, AR=2.9). **(B)** Peak energy plotted against the chemical adsorption of 1-hexanethiol for points A, B, and C in single AuNRs (25 nm × 73 nm, AR=2.9).

**
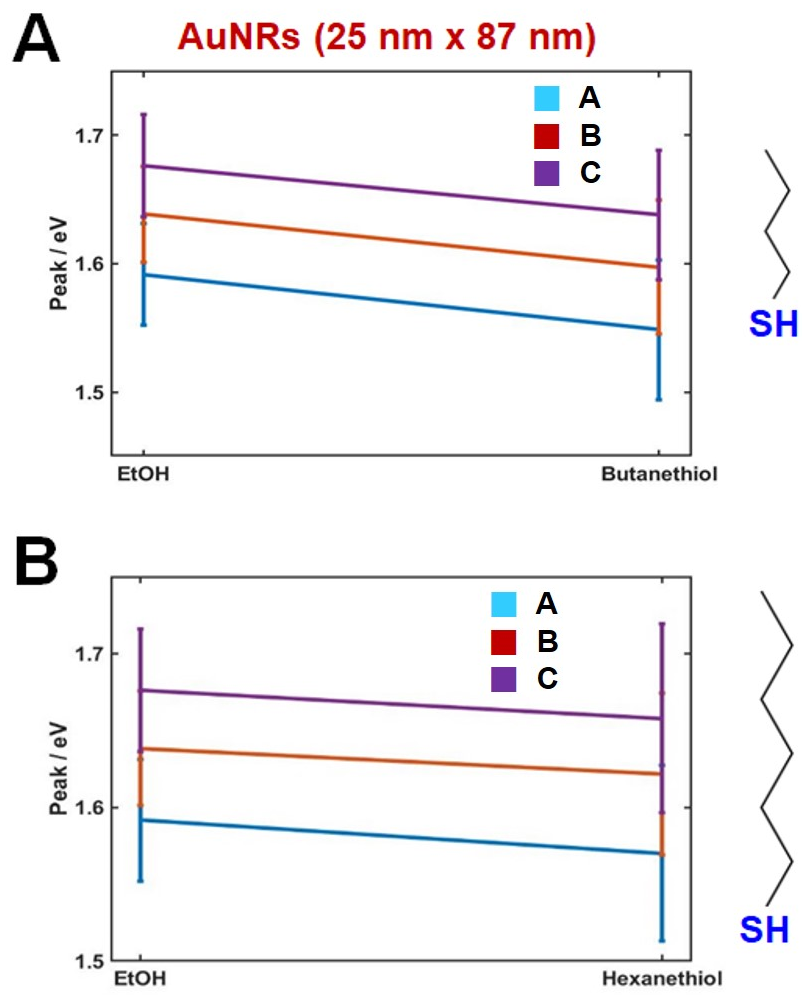
**

**Figure S5.** **(A)** Peak energy plotted against the chemical adsorption of 1-butanethiol for points A, B, and C in single AuNRs (25 nm × 87 nm, AR=3.5). **(B)** Peak energy plotted against the chemical adsorption of 1-hexanethiol for points A, B, and C in single AuNRs (25 nm × 87 nm, AR=3.5).
