# Supplementary material for: Localized surface plasmon resonance inflection points for improved detection of chemisorption of 1-alkanethiols under total internal reflection scattering microscopy
Source: Sci Rep. 2021 Jun 18;11:12902. doi: 10.1038/s41598-021-92410-w (PMC8213723; doi:10.1038/s41598-021-92410-w)
Supplement: Supplementary file 2 — Supplementary Information 2. [file 41598_2021_92410_MOESM2_ESM.pdf]

Supplementary Information

Localized Surface Plasmon Resonance Inflection Points for Improved Detection of Chemisorption of 1-alkanethiols under Total Internal Reflection Scattering Microscopy

Kyeong Rim Ryu,[a,#] Geun Wan Kim,[a,#] and Ji Won Ha[a,b*]

[a]Advanced Nano-Bio-Imaging and Spectroscopy Laboratory, Department of Chemistry, University of Ulsan, 93 Daehak-ro, Nam-gu, Ulsan 44610, Republic of Korea
[b]Energy Harvest-Storage Research Center (EHSRC), University of Ulsan, 93 Daehak-ro, Nam-gu, Ulsan 44610, Republic of Korea

[#]These authors contributed equally to this work.

[*]To whom correspondence should be addressed.
J. W. Ha
Phone: +82-52-712-8012
Fax: +82-52-712-8002
E-mail: jwha77@ulsan.ac.kr

This document contains supplementary figures (Figs. S1 to S5).

Supplementary Figures

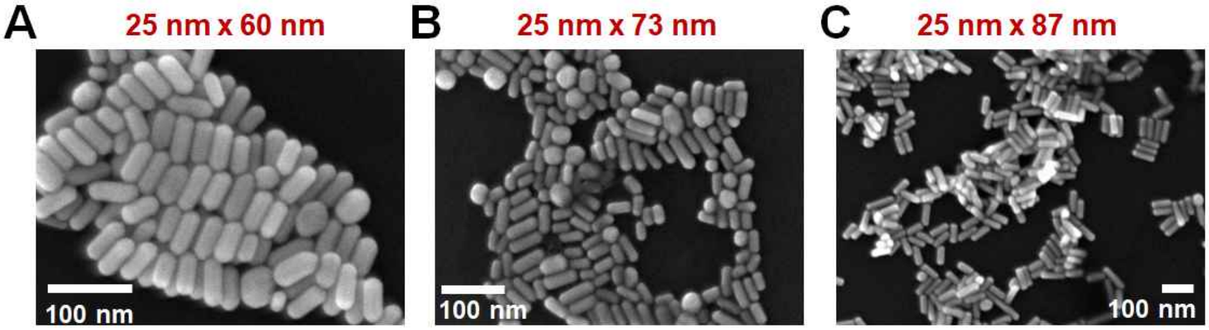

Figure S1. (A) SEM image of AuNRs with a AR of 2.4 (25 nm × 60 nm). **(B)** SEM image of AuNRs with a AR of 2.9 (25 nm × 73 nm). **(C)** SEM image of AuNRs with a AR of 3.5 (25 nm × 87 nm).

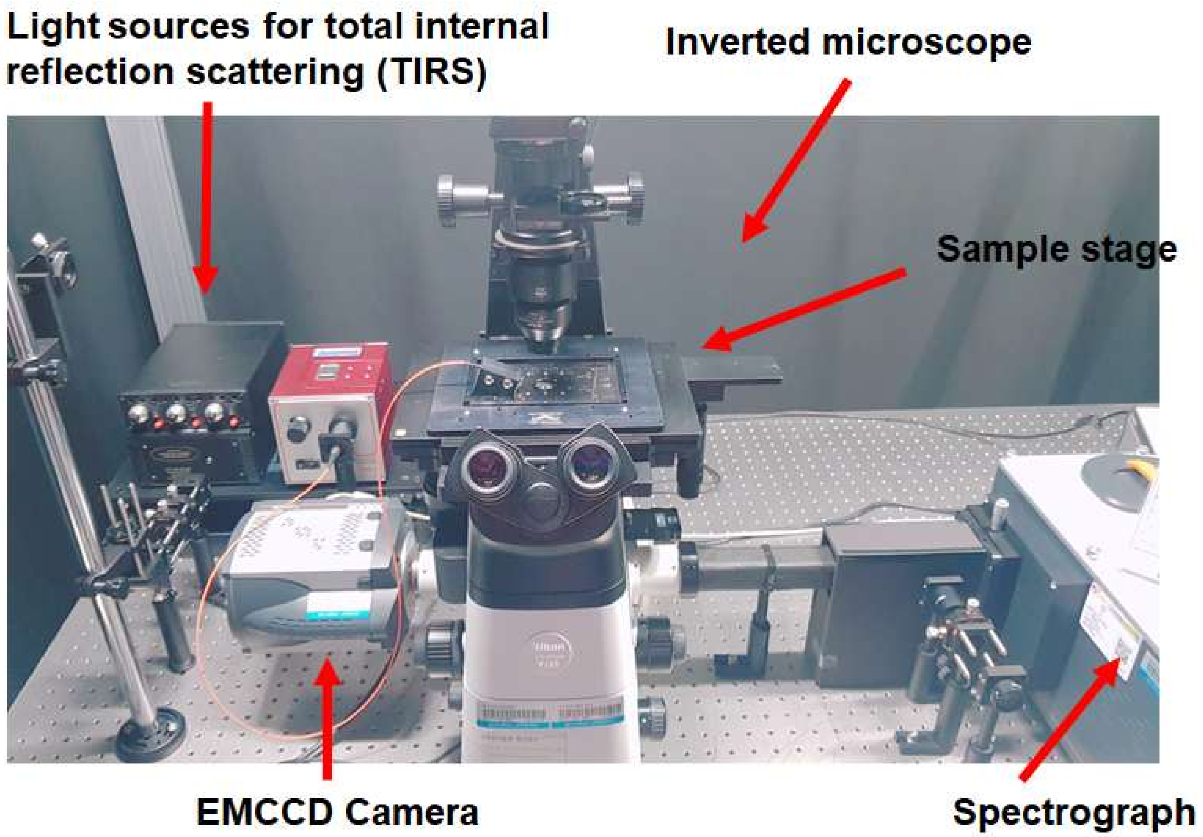

Figure S2. A photograph to show the experimental setup for single-particle TIRS microscopy and spectroscopy.

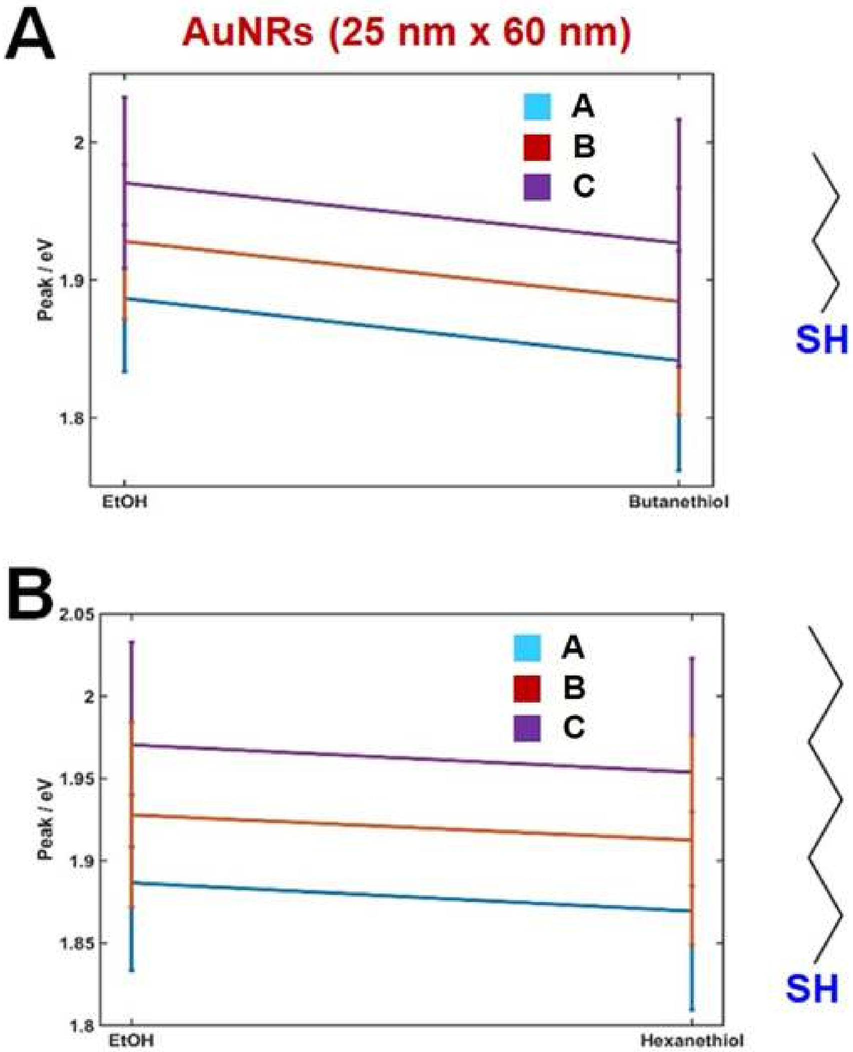

Figure S3. (A) Peak energy plotted against the chemical adsorption of 1-butanethiol for points A, B, and C in single AuNRs (25 nm × 60 nm, AR=2.4). **(B)** Peak energy plotted against the chemical adsorption of 1-hexanethiol for points A, B, and C in single AuNRs (25 nm × 60 nm, AR=2.4).

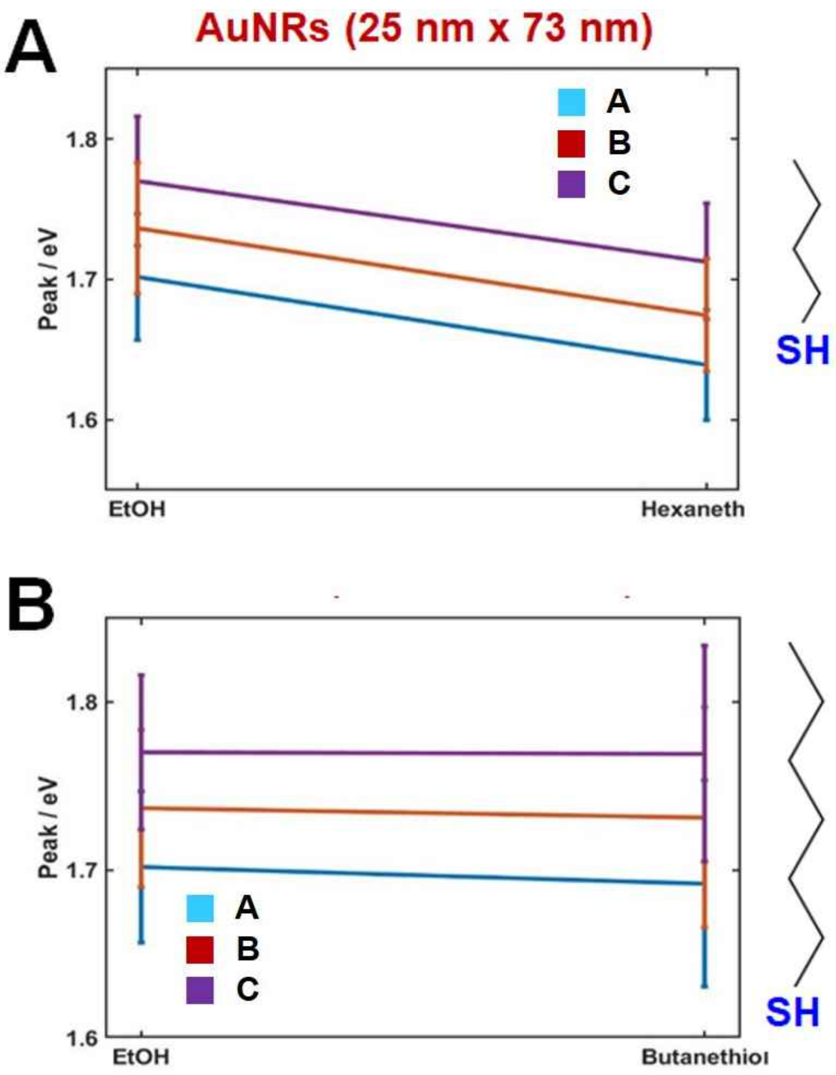

Figure S4. **(A)** Peak energy plotted against the chemical adsorption of 1-butanethiol for points A, B, and C in single AuNRs (25 nm × 73 nm, AR=2.9). **(B)** Peak energy plotted against the chemical adsorption of 1-hexanethiol for points A, B, and C in single AuNRs (25 nm × 73 nm, AR=2.9).

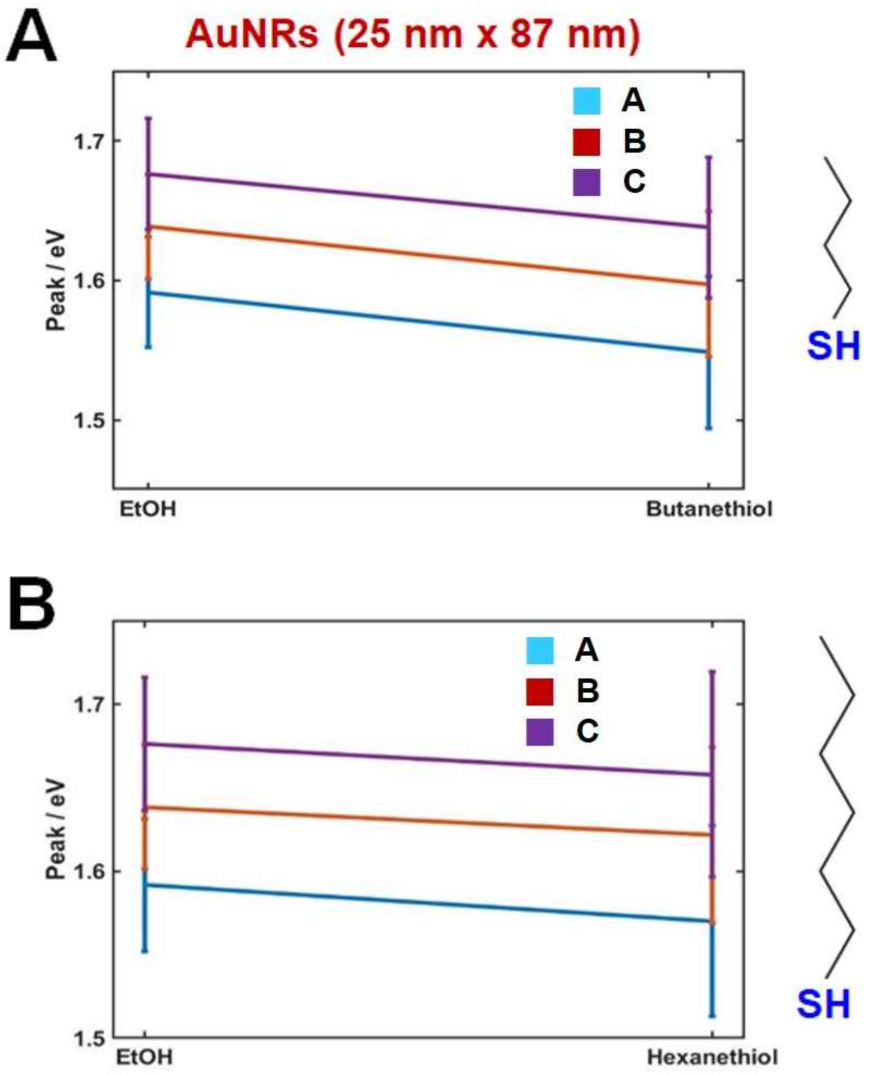

Figure S5. **(A)** Peak energy plotted against the chemical adsorption of 1-butanethiol for points A, B, and C in single AuNRs (25 nm × 87 nm, AR=3.5). **(B)** Peak energy plotted against the chemical adsorption of 1-hexanethiol for points A, B, and C in single AuNRs (25 nm × 87 nm, AR=3.5).
