# Supplementary material for: Localized surface plasmon resonance inflection points for improved detection of chemisorption of 1-alkanethiols under total internal reflection scattering microscopy
Source: Sci Rep. 2021 Jun 18;11:12902. doi: 10.1038/s41598-021-92410-w (PMC8213723; doi:10.1038/s41598-021-92410-w)

<Research Article>

Localized Surface Plasmon Resonance Inflection Points for Improved Detection of Chemisorption of 1-alkanethiols under Total Internal Reflection Scattering Microscopy

Kyeong Rim Ryu,[a,#] Geun Wan Kim,[a,#] and Ji Won Ha[a,b*]

[a]Advanced Nano-Bio-Imaging and Spectroscopy Laboratory, Department of Chemistry, University of Ulsan, 93 Daehak-ro, Nam-gu, Ulsan 44610, Republic of Korea
[b]Energy Harvest-Storage Research Center (EHSRC), University of Ulsan, 93 Daehak-ro, Nam-gu, Ulsan 44610, Republic of Korea

[#]These authors contributed equally to this work.

[*]To whom correspondence should be addressed.
J. W. Ha
Phone: +82-52-712-8012
Fax: +82-52-712-8002
E-mail: jwha77@ulsan.ac.kr

Abstract

Plasmonic gold nanoparticles are widely used in localized surface plasmon resonance (LSPR) sensing. When target molecules adsorb to the nanoparticles, they induce a shift in the LSPR scattering spectrum. In conventional LSPR sensing, this shift is monitored at the maximum of the LSPR scattering peak. Herein, we describe the sensitivity of detecting chemisorption of 1-alkanethiols with different chain lengths (1-butanethiol and 1-haxanethiol) on single gold nanorods (AuNRs) of fixed diameter (25 nm) and three different aspect ratios under a total internal reflection scattering microscope. For single AuNRs of all sizes, the inflection point (IF) at the long-wavelength side (or low-energy side) of the LSPR scattering peak showed higher detection sensitivity than the traditionally used peak maximum. The improved sensitivity can be ascribed to the shape change of the LSPR peak when the local refractive index is increased by chemisorption. Our results demonstrate the usefulness of tracking the curvature shapes by monitoring the homogeneous LSPR IF at the red side of the scattering spectrum of single AuNRs.

Keywords: Gold nanorods, LSPR sensing, Inflection points, Total internal reflection scattering microscopy, Single-particle spectroscopy

Introduction

Plasmonic gold nanoparticles (AuNPs) have attracted considerable attention in biosensor developments[1] because their localized surface plasmon resonance (LSPR) effect confers unique optical properties[2-5]; moreover, AuNPs are biocompatible[6], chemically stable[7], and are conveniently surface-modified with organic and biological molecules[8,9]. Conventional LSPR biosensors employ AuNPs functionalized with receptors that confer specific binding abilities to target molecules. When a target molecule adsorbs on the nanoparticle surface, the LSPR peak is shifted and dampened[10]. Thus, by monitoring the shifts and broadening of the peaks in the LSPR spectrum of AuNPs[11], we can detect the presence of target molecules[12].

Improving the sensitivity of LSPR sensors has been the goal of many researchers. In one approach, plasmonic nanoparticles with high polarizability are synthesized, such as nanorods, nanostars, nanoshells, nanoholes, and alloy nanoparticles[13]. Another approach uses a simple mathematical method that tracks the shifts at the inflection points (IFs) of the LSPR spectrum. LSPR-IF tracking is a complementary method that further improves the detection sensitivity of LSPR biosensors. Chen *et al.* showed that the refractive index (RI) sensitivity was higher at the IFs located at the long-wavelength (low-energy) side of the LSPR extinction peak than at the short-wavelength side[13-15]. Very recently, the RI sensitivities of homogeneous LSPR IFs have been reported for a range of single AuNPs: gold nanocubes, gold bipyramids, and bimetallic gold nanorods (AuNRs). The LSPR IFs exhibit a higher RI sensitivity than the frequency shifts of their counterpart LSPR peaks[16-18]. However, at present, understanding the response from the change in the local RI induced by molecular binding to AuNPs is still very limited at the single-particle level. Furthermore, there have been no studies to characterize the detection sensitivity at the LSPR IFs of single AuNRs induced by chemisorption of 1-alkanethiols with different

carbon-chain lengths. Thus, it is necessary to better understand the effect of carbon-chain length on the detection sensitivity at the LSPR IFs in single AuNRs.

In addition, conventional dark-field (DF) spectroscopy has been widely used so far to investigate the RI sensitivities of homogeneous LSPR IFs of single AuNPs. However, total internal reflection scattering (TIRS) microscopy[19-21] based on evanescent field illumination at the interface has rarely been used for the sensitivity of LSPR sensors.

In the present study, we evaluate the detection sensitivity of the LSPR IFs in the homogeneous scattering spectra of single AuNRs to chemical adsorption of 1-alkanethiols with different carbon-chain lengths. Single AuNRs of constant diameter (25 nm) and three different aspect ratios (ARs) were employed for this purpose. The results indicate that by tracking the homogeneous LSPR IF at the red side, we can sensitively detect the chemisorption of thiol molecules on single AuNRs.

Results and Discussion

Experiments were performed on AuNRs with a fixed diameter (25 nm) and three different ARs (2.4, 2.9 and 3.5). The sizes and shapes of the AuNRs were characterized by scanning electron microscopy (SEM), and the images are displayed in panels A−C of Fig. 1 (additional SEM images are provided in Fig. S1). Fig. 1D shows the extinction spectra of the AuNRs dispersed in water, obtained in a UV−Vis spectrophotometer (Fig. 1D). As the AR of the AuNRs increased, the longitudinal LSPR peak was red-shifted. However, heterogeneity issues limit the usefulness of ensemble-level measurements, and single-particle measurements are required for a deeper understanding of their characteristic optical properties.

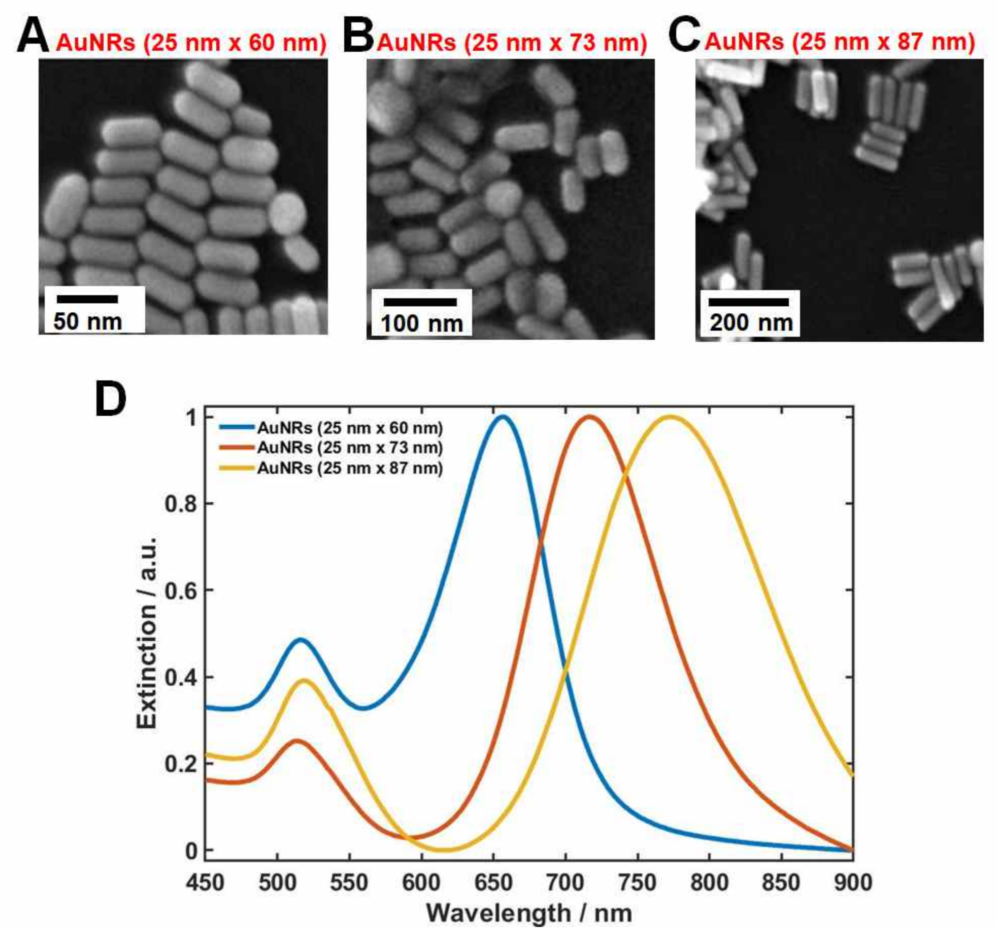

Figure 1. (A)−(C) SEM images of AuNRs with different aspect ratios: **(A)** 2.4 (25 nm × 60 nm), **(B)** 2.9 (25 nm × 73 nm), **(C)** 3.5 (25 nm × 87 nm); **(D)** Overlaid extinction spectra of AuNRs with ARs of 2.4, 2.9, and 3.5 measured in water

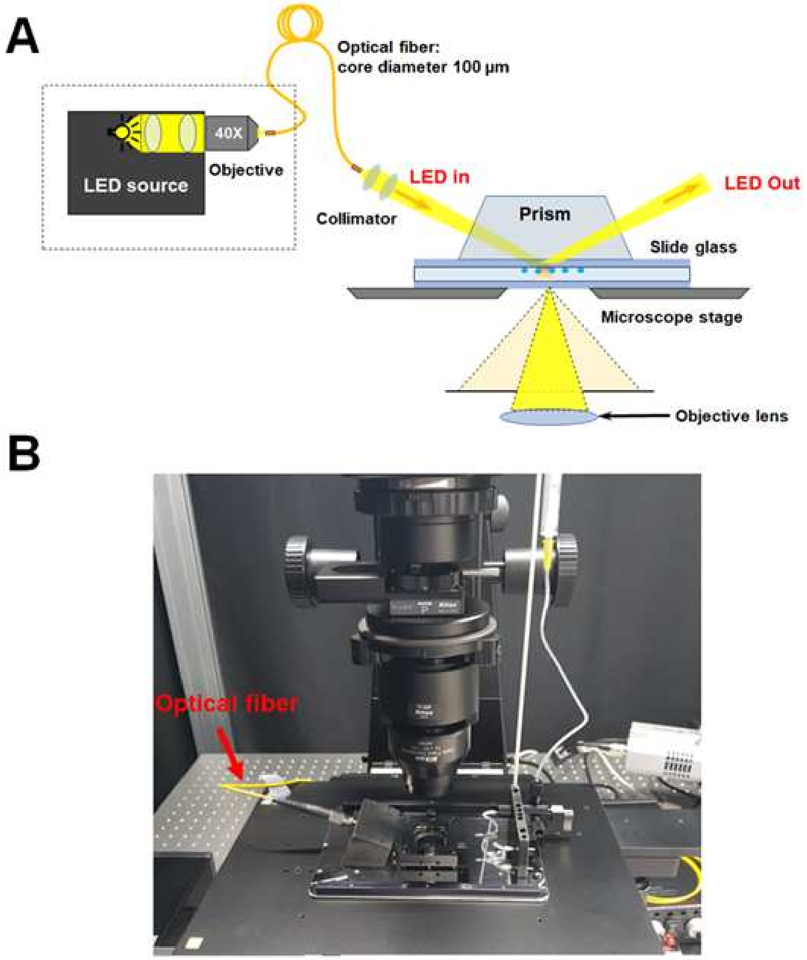

Figure 2. (A) Schematic and **(B)** photograph of the experimental setup for TIRS microscopy and spectroscopy

The optical properties of the AuNRs were characterized by TIRS microscopy and single-particle spectroscopy (see Fig. 2)[19,22]. The experimental setup for single-particle TIRS microscopy and spectroscopy is shown in Figs. 2B and S2. For TIRS scattering measurements,

aqueous solutions of the AuNRs were drop-casted on a pre-cleaned glass slide, then illuminated with LED light delivered through an optical fiber (Fig. 2). Fig. 3A shows the TIRS scattering images of three single AuNRs with an AR of 2.4 (25 nm × 60 nm), and Fig. 3B shows their corresponding scattering spectra. In the single-particle scattering spectra of AuNRs in ethanol, a longitudinal LSPR peak was observed around 656 nm (1.89 eV). In the TIRS scattering image of single AuNRs with an AR of 2.9 (25 nm × 73 nm; Fig. 3C), the longitudinal LSPR peak was redshifted to approximately 716 nm (1.73 eV) (Fig. 3D). The longitudinal LSPR peak of single AuNRs with an AR of 3.5 (25 nm × 87 nm) was further redshifted to 785 nm (1.58 eV) (Figs. 3E and 3F).

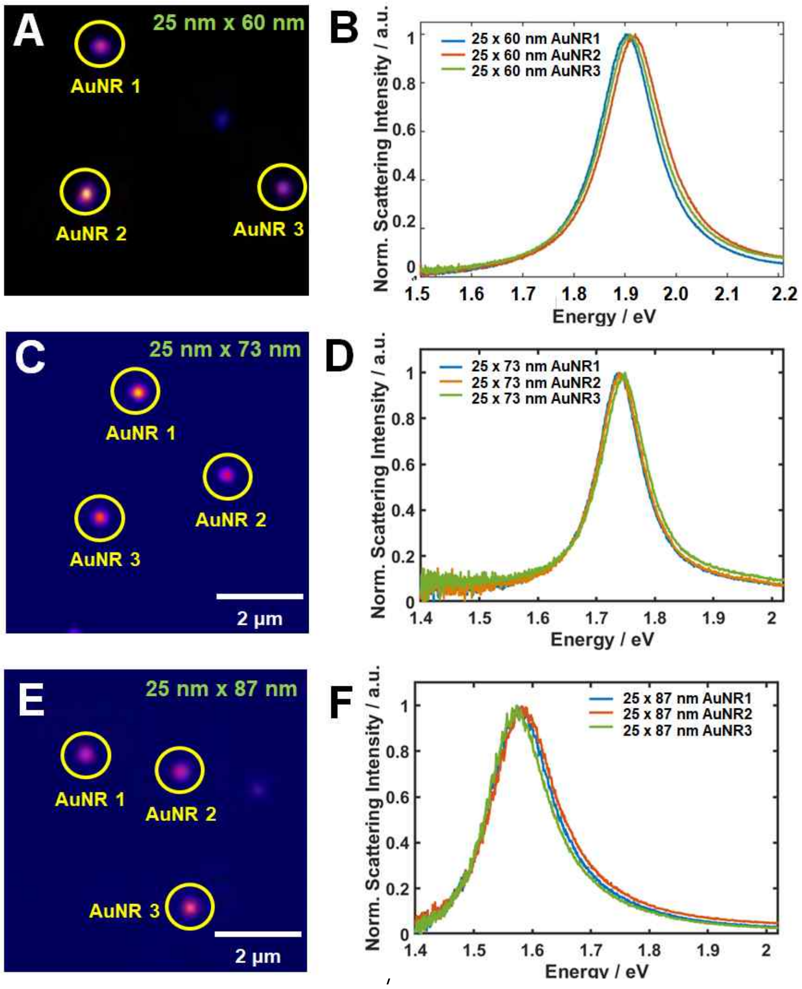

Figure 3. (A) TIRS scattering images of single AuNRs (25 nm × 60 nm, AR = 2.4) illuminated by white light; **(B)** Scattering spectra of the single AuNRs enclosed by the yellow circle in (**A**); **(C)** and **(D):** As for **(A)** and **(B)**, respectively, but for single AuNRs with AR = 2.9; (25 nm × 73 nm); **(E)** and **(F):** As for **(A)** and **(B)**, respectively, but for single AuNRs with AR = 3.5 (25 nm × 87 nm)

Thiol reactions with Au have been widely exploited in both the synthesis and surface modification of Au nanoparticles[23,24]. For this reason, we employed thiol molecules as the adsorbate molecules to AuNRs. Fig. 4A shows the structures of 1-alkanethiols with two different carbon-chain lengths (1-butanethiol and 1-hexanethiol) used in the detection sensitivity analysis of the LSPR IFs in the homogeneous scattering spectra of single AuNRs. As depicted in Fig. 4B, thiol molecules can strongly bond with the Au surface through soft−soft Au−sulfur interactions.[16,25]

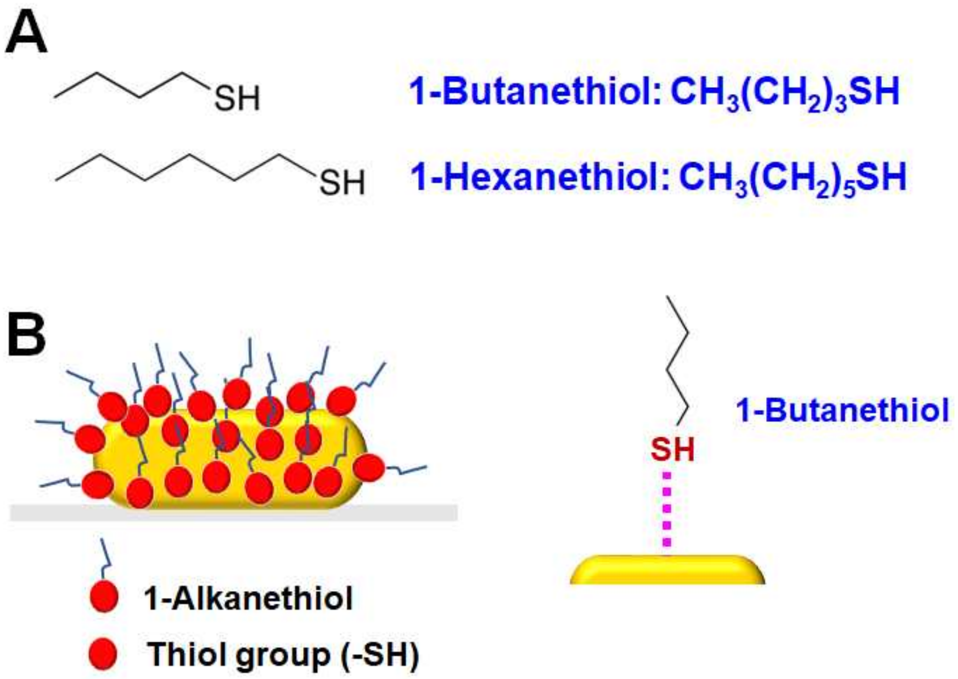

Figure 4. (A) Chemical structure of 1-butanethiol and 1-hexanethiol used as the adsorbate molecules; **(B)** Schematic of a single AuNR interacting with a 1-alkanethiol through a strong Au−sulfur bond

First, we investigated the LSPR peak shifts caused by chemical interactions between the 1-alkanethiols in ethanol and single AuNRs (25 nm × 60 nm; AR = 2.4). The first, second, and third rows of Fig. 5 show the scattering spectra of single AuNRs with an AR of 2.4 (25 nm × 60 nm) and their first- and second order derivatives, respectively. The maxima of the LSPR scattering peaks (labeled "B" in Fig. 5) appeared at 1.91 eV for bare AuNR (no thiol; Fig. 5A), 1.75 eV for AuNR chemisorbed to 1-butanethiol (Fig. 5B), and 1.85 eV for AuNR chemisorbed to 1-hexanethiol (Fig. 5C). Furthermore, second-order derivatives were obtained at the IFs in the LSPR scattering spectra (third row) of bare AuNR and AuNR chemisorbed to 1-alkanethiols (1-butanethiol and 1-hexanethiol). Note that the LSPR IFs coincide with the local maxima/minima of the first-order derivatives. As observed in the first order derivative, the LSPR maxima (labeled "B") are the apparent critical points in the LSPR scattering spectra of AuNRs, as their values in the first-order derivative spectra are zero in each case. Moreover, the characteristic shapes of the first- and second-order derivatives of the LSPR scattering spectra of single AuNRs matched those of a previous report on the LSPR IFs of AuNPs[16,17]. As evidenced from their curvatures, the LSPR scattering curves and their second-order derivatives were even functions and symmetrical about the axis of intensity, whereas the first-order derivatives were odd functions and symmetrical about the axis of photon energy. Fig. S3 plots the energy peaks in points A, B, and C against the adsorption of 1-butanethiol and 1-hexanethiol on the Au surface in ethanol. IF A, located at the long-wavelength (low-energy) side of the LSPR scattering peak, more

sensitively detected both 1-alkanethols (1-butanethiol and 1-hexanethiol) than the traditionally used LSPR peak maximum (B) (see Figs. 5D and 5E).

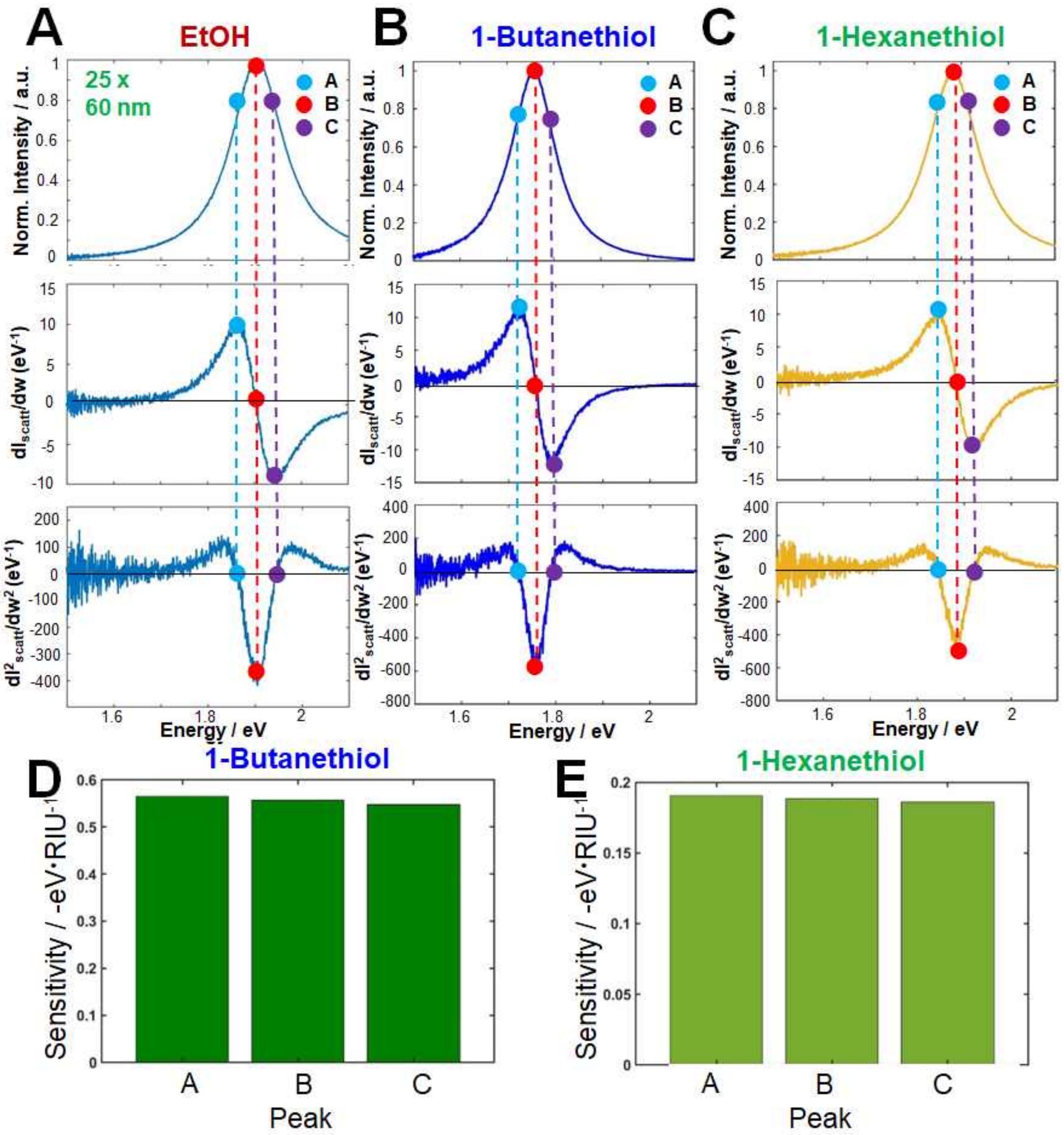

Figure 5. Inflection-point method for single-particle LSPR scattering sensing with AuNRs (25 nm × 60 nm, AR = 2.4) in the presence of 1-alkanethiols (1-butanethiol and 1-hexanethiol) in ethanol: **(A−C)** LSPR scattering efficiencies (first row), and their first-order (second row) and second-order (third row) derivatives; **(D, E)** Detection sensitivities of peak shifts in A, B and C.

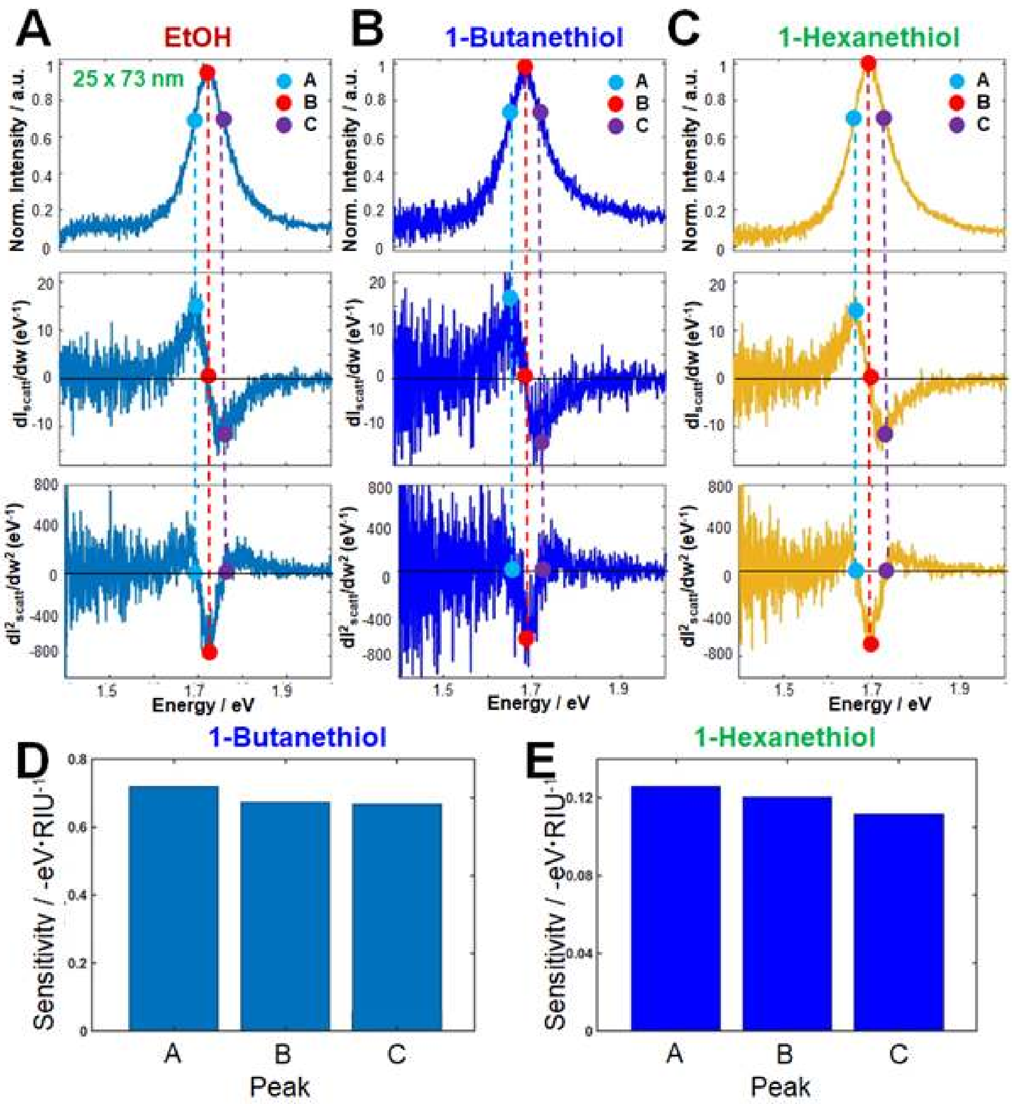

Figure 6. Inflection-point method for single-particle LSPR scattering sensing with AuNRs (25 nm × 73 nm, AR = 2.9) in the presence of 1-alkanethiols (1-butanethiol and 1-hexanethiol) in ethanol: **(A−C)** LSPR scattering efficiencies (first row), and their first-order (second row), and second-order (third row) derivatives; **(D, E)** Detection sensitivities of peak shifts in A, B and C.

To check whether IF A indeed has a higher detection sensitivity than the peak maximum in the LSPR, we observed single AuNRs with a higher AR (25 nm × 73 nm; AR = 2.9). Panels A, B, and C of Fig. 6 show the scattering spectra of single AuNRs (A = 2.9) and their corresponding

first and second order derivatives, respectively. Columns A, B, and C of this figure correspond to bare AuNR (no thiol), AuNR chemisorbed with 1-butanethiol, and AuNR chemisorbed with 1-hexanethiol, respectively. The maxima of the LSPR scattering peaks (again labeled B) in the environments of panels A, B, and C were 1.72, 1.68, and 1.69 eV, respectively. Fig. S4 plots the energy peaks in points A, B, and C against the adsorptions of 1-butanethiol and 1-hexanethiol on the Au surface. IF A, located at the long-wavelength (low-energy) side of the LSPR scattering peak, again exhibited higher sensitivity than the LSPR peak maxima (B) for 1-alkanethol detection (Figs. 6D and 6E), consistent with the results of single AuNRs with lower AR (2.4; see Fig. 5).

Finally, we obtained the scattering spectra of single AuNRs with the highest AR (25 nm × 87 nm; AR = 3.5) and their corresponding first and second order derivatives. The results are displayed in panels A, B, and C of Fig. 7, respectively. In the LSPR scattering spectra of the bare AuNRs, AuNRs adsorbed to 1-butanethiol, and AuNRs adsorbed to 1-hexanethiol, the peak maxima appeared at 1.67, 1.58, and 1.60 eV. respectively. Fig. S5 plots the energy peaks in points A, B, and C against the adsorptions of 1-butanethiol and 1-hexanethiol on the Au surface. Similarly to Figs. 5 and 6, the highest detection sensitivity to 1-alkanethiols was found at IF A of the LSPR (Figs. 7D and 7E). Thus, regardless of AR and adsorbate type, the sensitivity of single AuNRs to chemisorption of 1-alkanethols is higher at IF A (long-wavelength side) of the LSPR than at the maximum of the counterpart peak, which is widely used in LSPR sensing. The improved sensitivity at IF A can be ascribed to the shape change of the LSPR peak when the local refractive index is altered by chemisorption[13,15]. Therefore, tracking the curvature shapes through the homogeneous LSPR IF at the red side of the scattering peak (rather than the shifts of

their counterpart peak maxima) can enhance the detection sensitivities of differently sized AuNRs.

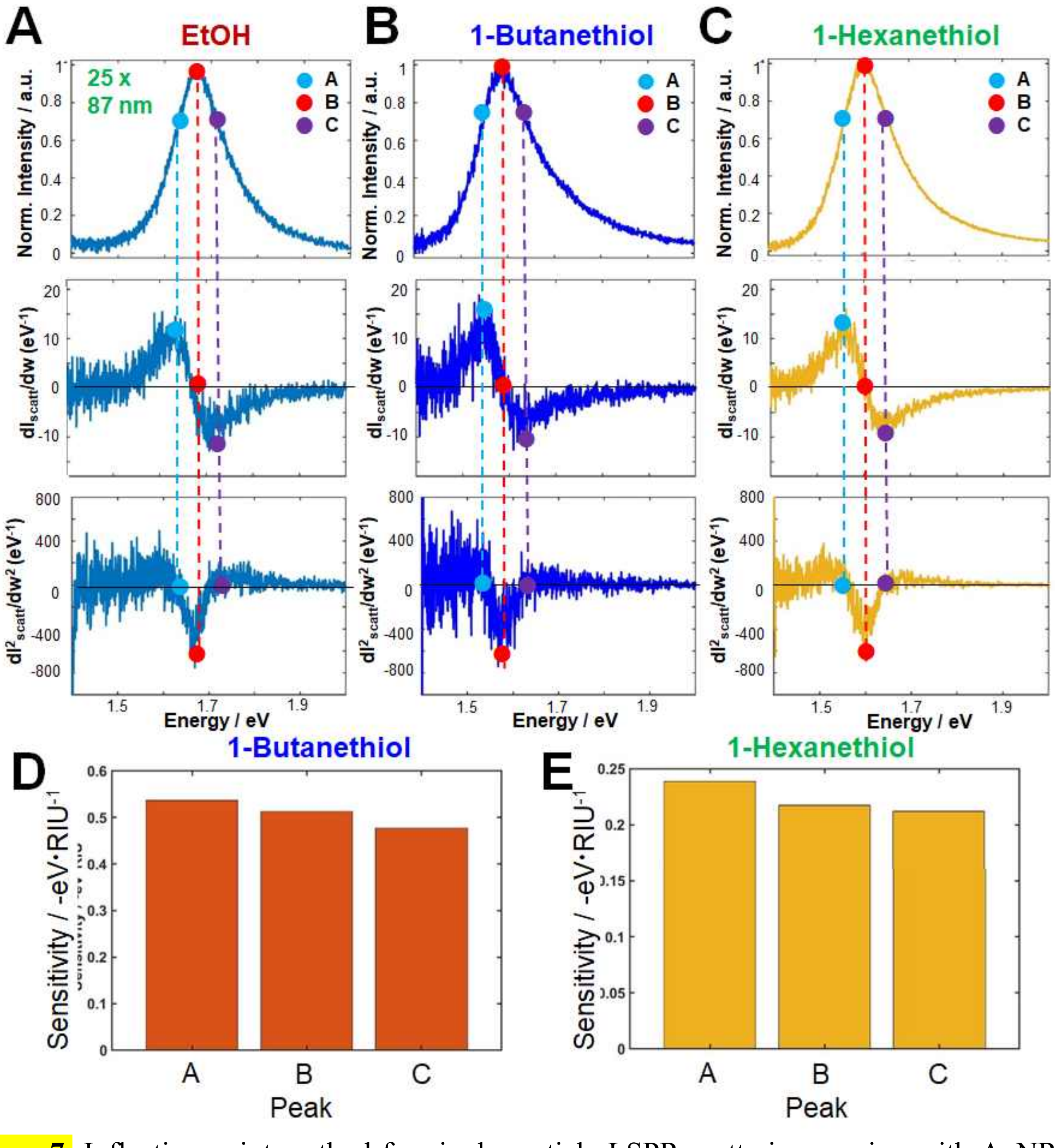

Figure 7. Inflection-point method for single-particle LSPR scattering sensing with AuNRs (25 nm × 87 nm, AR = 3.5) in the presence of 1-alkanethiols (1-butanethiol and 1-hexanethiol) in ethanol: **(A−C)** LSPR scattering efficiencies (first row), and their first-order (second row), and second-order (third row) derivatives; **(D, E)** Detection sensitivities of peak shifts in A, B and C.

Conclusions

We demonstrated the detection sensitivity of single AuNRs for chemisorption of 1-alkanethiols with two different chain lengths. Single AuNRs with the same diameter and different ARs (2.4, 2.9, and 3.5) were employed, and single-particle TIRS microscopy and spectroscopy techniques were used to examine them. In the chemisorption of both 1-butanethiol and 1-haxanethiol, IF A at the long-wavelength side of the homogeneous LSPR scattering peak more sensitively detected the absorbates than the conventionally used peak maximum of the LSPR. The detection improvement was consistent for the AuNRs with three different ARs. The higher sensitivity of IF A at the red side was attributed to the shape change of the LSPR scattering peak when the local refractive index was increased after chemisorption. Therefore, this study provided a deeper understanding of improved detection sensitivity of 1-alkanethiol adsorption at homogeneous LSPR IFs in single AuNRs under TIRS microscopy and spectroscopy.

Methods

Chemicals and Materials. Cetyltrimethylammonium bromide (CTAB)-stabilized AuNRs of three different sizes (25 nm × 60 nm, 25 nm × 73 nm, 25 nm × 87 nm) were purchased from Nanopartz (Loveland, CO, USA). 1-alkanethiols (1-butanethiol and 1-hexanethiol) were obtained from Sigma-Aldrich (St. Louis, MO, USA). Immersion oil was also purchased from Sigma-Aldrich.

Characterization of AuNRs. The shapes and sizes of the AuNRs were determined by scanning electron microscopy (SEM, JSM-6500, JEOL, Japan). The LSPR extinction spectra of the AuNRs dispersed in water were measured using a Varian Carry 300 UV−Vis spectrophotometer (Agilent, USA).

Sample Preparation for Single-Particle Study. Microscope cover glasses were cleaned by sonicating in methanol for 10 min, followed by acetone for 10 min. The solution containing the AuNRs was diluted to the proper concentration and sonicated for 10 min to prevent aggregation of the AuNRs. Samples were prepared by drop-casting the diluted AuNR solution onto the glass slides. The concentration of Au nanoparticles deposited on the glass slide was adjusted to approximately 1 μm^{-2} to facilitate the measurement of single particles without inter-particle LSPR coupling.

Total Internal Reflection Scattering Microscopy. Scattering images of the AuNRs were collected on a home-built TIRS microscope. The TIRS microscope was excited by LED lamps through an inverted Nikon microscope (Nikon Eclipse Ti-2). For TIRS microscopy, the beam

from the LED source passed through an optical fiber with a core diameter of 100 μm. Collimating lenses were placed at both ends of the optical fiber. Incident light was directed into the samples through a glass prism at fixed incident angle (70°). The microscope utilized a Nikon Plan Fluor oil objective [100×, numerical aperture (NA) = 0.5–1.3]. Here, the NA of the objective was maintained at 0.7. The DF and TIRS images of the AuNRs were recorded by an Andor iXon^{EM}+CCD camera (iXon897). The collected images were analyzed by ImageJ and Matlab.

Single-Particle Scattering Spectroscopy. Scattering spectra were acquired with a spectrometer (Andor, Kymer328i-A) and a CCD camera (Andor, Newton920). A relay lens system [a Newton lens system with two lenses (f = 7.5 cm)] was inserted between the microscope and the spectrometer system. When acquiring the spectrum of a single AuNR, the relay lens system was moved perpendicularly to the optical axis on a translational stage. In this setup, only the light scattered from the target AuNR particles was passed to the spectrometer. The light scattered by the AuNRs was dispersed through a grating (300 /mm) and detected by a charge-coupled device camera. The background was measured from a region devoid of particles.

Acknowledgment

This study was supported by two National Research Foundation of Korea (NRF) grants funded by the Korean government (MSIP) (No. 2018R1C1B3001154 and No. 2019R1A6A1A11053838).

Author Contributions

K.R.R. and G.W.K. performed the single-particle scattering measurements. K.R.R., G.W.K, and J.W.H. analyzed the data, and J.W.H wrote the paper.

Additional Information

Supplementary Information: Additional supplementary figures (Figs. S1 to S5) as noted in the text

Competing Interests: The authors declare no competing interests.

Publisher’s note: Springer Nature remains neutral with regard to jurisdictional claims in published maps and institutional affiliations.

References

1 Jans, H. & Huo, Q. Gold nanoparticle-enabled biological and chemical detection and analysis. *Chemical Society Reviews* **41**, 2849-2866, doi:10.1039/C1CS15280G (2012).

2 Nehl, C. L., Liao, H. & Hafner, J. H. Optical properties of star-shaped gold nanoparticles. *Nano letters* **6**, 683-688 (2006).

3 Nehl, C. L. & Hafner, J. H. Shape-dependent plasmon resonances of gold nanoparticles. *Journal of Materials Chemistry* **18**, 2415-2419 (2008).

4 Sosa, I. O., Noguez, C. & Barrera, R. G. Optical properties of metal nanoparticles with arbitrary shapes. *The Journal of Physical Chemistry B* **107**, 6269-6275 (2003).

5 Hu, M. *et al.* Gold nanostructures: engineering their plasmonic properties for biomedical applications. *Chemical Society Reviews* **35**, 1084-1094 (2006).

6 Jazayeri, M. H., Amani, H., Pourfatollah, A. A., Pazoki-Toroudi, H. & Sedighimoghaddam, B. Various methods of gold nanoparticles (GNPs) conjugation to antibodies. *Sensing and Bio-Sensing Research* **9**, 17-22, doi:https://doi.org/10.1016/j.sbsr.2016.04.002 (2016).

7 Lee, D.-E. *et al.* Multifunctional nanoparticles for multimodal imaging and theragnosis. *Chemical Society Reviews* **41**, 2656-2672, doi:10.1039/C2CS15261D (2012).

8 Chen, Y., Xianyu, Y. & Jiang, X. Surface Modification of Gold Nanoparticles with Small Molecules for Biochemical Analysis. *Accounts of chemical research* **50**, 310-319, doi:10.1021/acs.accounts.6b00506 (2017).

9 Sperling, R. A. & Parak, W. J. Surface modification, functionalization and bioconjugation of colloidal inorganic nanoparticles. *Philosophical Transactions of the Royal Society A: Mathematical, Physical and Engineering Sciences* **368**, 1333 (2010).

10 Nusz, G. J., Curry, A. C., Marinakos, S. M., Wax, A. & Chilkoti, A. Rational Selection of Gold Nanorod Geometry for Label-Free Plasmonic Biosensors. *ACS Nano* **3**, 795-806, doi:10.1021/nn8006465 (2009).

11 Yang, S., Wu, T., Zhao, X., Li, X. & Tan, W. The Optical Property of Core-Shell Nanosensors and Detection of Atrazine Based on Localized Surface Plasmon Resonance (LSPR) Sensing. *Sensors (Basel, Switzerland)* **14**, 13273-13284, doi:10.3390/s140713273 (2014).

12 Hu, M. *et al.* Dark-field microscopy studies of single metal nanoparticles: understanding the factors that influence the linewidth of the localized surface plasmon resonance. *Journal of materials chemistry* **18**, 1949-1960, doi:10.1039/b714759g (2008).

13 Chen, P., Tran, N. T., Wen, X., Xiong, Q. & Liedberg, B. Inflection Point of the Localized Surface Plasmon Resonance Peak: A General Method to Improve the Sensitivity. *ACS sensors* **2**, 235-242 (2017).

14 Chen, P., Tran, N. T., Wen, X., Xiong, Q. & Liedberg, B. Inflection Point of the Localized Surface Plasmon Resonance Peak: A General Method to Improve the Sensitivity. *ACS Sens* **2**, 235-242, doi:10.1021/acssensors.6b00633 (2017).

15 Chen, P. & Liedberg, B. Curvature of the localized surface plasmon resonance peak. *Analytical chemistry* **86**, 7399-7405 (2014).

16 Tsalu, P. V., Kim, G. W., Hong, J. W. & Ha, J. W. Homogeneous localized surface plasmon resonance inflection points for enhanced sensitivity and tracking plasmon damping in single gold bipyramids. *Nanoscale* **10**, 12554-12563, doi:10.1039/C8NR03311K (2018).

17 Jeon, H. B., Tsalu, P. V. & Ha, J. W. Shape Effect on the Refractive Index Sensitivity at Localized Surface Plasmon Resonance Inflection Points of Single Gold Nanocubes with Vertices. *Scientific Reports* **9**, 13635, doi:10.1038/s41598-019-50032-3 (2019).

18 Ryu, K. R. & Ha, J. W. Influence of shell thickness on the refractive index sensitivity of localized surface plasmon resonance inflection points in silver-coated gold nanorods. *RSC Advances* **10**, 16827-16831, doi:10.1039/D0RA02691C (2020).

19 Lee, J., Kim, G. W. & Ha, J. W. Single-particle spectroscopy and defocused imaging of anisotropic gold nanorods by total internal reflection scattering microscopy. *Analyst* **145**, 6038-6044, doi:10.1039/D0AN01071E (2020).

20 Balevicius, Z. *et al.* Total internal reflection ellipsometry of metal–organic compound structures modified with gold nanoparticles. *Thin Solid Films* **519**, 2959-2962, doi:https://doi.org/10.1016/j.tsf.2010.12.170 (2011).

21 Oates, T. W. H., Wormeester, H. & Arwin, H. Characterization of plasmonic effects in thin films and metamaterials using spectroscopic ellipsometry. *Progress in Surface Science* **86**, 328-376, doi:https://doi.org/10.1016/j.progsurf.2011.08.004 (2011).

22 Jeon, H. B. & Ha, J. W. Single-Particle Study: Plasmon Damping of Gold Nanocubes with Vertices by Adsorbate Molecules. *Bulletin of the Korean Chemical Society* **39**, 1117-1119 (2018).

23 Mazloomi-Rezvani, M., Salami-Kalajahi, M., Roghani-Mamaqani, H. & Pirayesh, A. Effect of surface modification with various thiol compounds on colloidal stability of gold nanoparticles. *Applied Organometallic Chemistry* **32**, e4079, doi:https://doi.org/10.1002/aoc.4079 (2018).

24 Gao, J., Huang, X., Liu, H., Zan, F. & Ren, J. Colloidal Stability of Gold Nanoparticles Modified with Thiol Compounds: Bioconjugation and Application in Cancer Cell Imaging. *Langmuir : the ACS journal of surfaces and colloids* **28**, 4464-4471, doi:10.1021/la204289k (2012).

25 Lee, S. Y. *et al.* Tuning Chemical Interface Damping: Interfacial Electronic Effects of Adsorbate Molecules and Sharp Tips of Single Gold Bipyramids. *Nano Letters* **19**, 2568-2574, doi:10.1021/acs.nanolett.9b00338 (2019).

Graphical Abstract

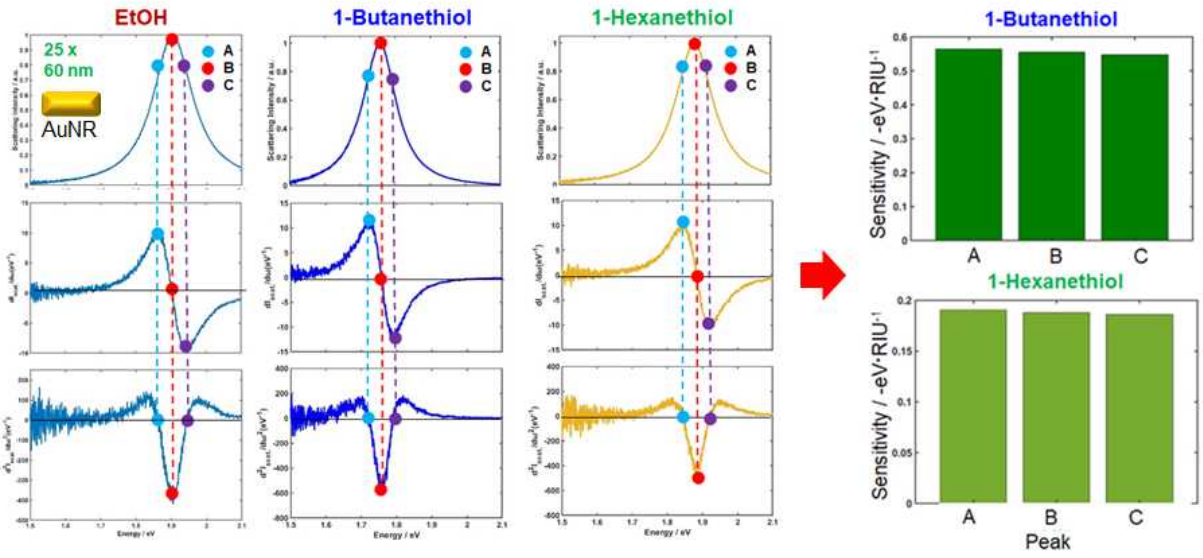

Supplement: Supplementary file 3 — Supplementary Information 3. [file 41598_2021_92410_MOESM3_ESM.pdf]
